# Exploring the feasibility and acceptability of Continuous Glucose Monitoring among people with type 1 diabetes and healthcare providers in South Africa’s public sector: A qualitative study

**DOI:** 10.1371/journal.pone.0352590

**Published:** 2026-07-16

**Authors:** Lorrein Muhwava, Elena Marbán-Castro, Mpho Silima, Maria Karsas, Tanja Kemp, Johane Freitas, Yvonne Kamau, Beatrice Vetter, Cathy Haldane, Paul Rheeder, Michelle Carrihill, Joel A. Dave, Sonjelle Shilton

**Affiliations:** 1 FIND. Global Health Campus, Chem. du Pommier, Le Grand-Saconnex, Switzerland; 2 Department of Internal Medicine and Paediatrics. Steve Biko Academic Hospital, University of Pretoria. Corner of Steve Biko and Malan Street. Gezina, Pretoria, South Africa; 3 Red Cross Children’s Hospital, Paediatric Clinic, Rondebosch, Cape Town, South Africa; 4 Faculty of Health Sciences, University of Cape Town, South Africa; 5 Division of Endocrinology, Groote Schuur Hospital, Observatory, Cape Town, South Africa; Kwara State University, NIGERIA

## Abstract

**Background:**

Continuous Glucose Monitoring (CGM) is an advancement in diabetes management, offering real-time insights into blood glucose levels and facilitating decision-making around diabetes care. However, its adoption in low- and middle-income countries (LMICs) remains low. This study explored the perceptions and experiences of CGM use among people living with type 1 diabetes (T1D), their caregivers, and healthcare providers (HCPs) in the public sector in South Africa.

**Methods:**

Convenience sampling was used to recruit participants from the ACCEDE study – a pragmatic randomised controlled trial on CGM use among people living with T1D in South Africa. Between July 2024 and February 2025, we conducted focus group discussions (FGDs) with recipients of care and caregivers and semi-structured interviews (SSIs) with HCPs across study sites in Cape Town and Pretoria. All FGDs and SSIs were audio-recorded, transcribed, and analysed using MAXQDA 2022. Data analysis followed thematic content analysis, and the findings presented according to the socioecological framework.

**Results:**

A total of 107 participants, including 75 people living with T1D, 17 caregivers, and 15 HCPs, were included in the study. CGM was generally perceived as acceptable due to convenience, reduced need for finger-prick testing, and enhanced understanding of glucose patterns. Feasibility was enhanced by ease of use, access to training, and social support. Key barriers included cost, limited awareness, and socio-economic constraints such as food insecurity and lack of access to transport.

**Conclusion:**

CGMs are both feasible and acceptable within South Africa’s public healthcare context. However, integration requires a coordinated, multi-level response, strengthening provider and patient awareness, knowledge, adequately resourced multi-disciplinary diabetes care teams, and addressing affordability through supportive health policies.

## Introduction

Continuous Glucose Monitoring (CGM) is a significant advancement in diabetes management, offering real-time insights into blood glucose levels and facilitating informed decision-making around diabetes care [1,2]. CGMs reduce the burden of frequent finger-prick testing and support personalised diabetes management through data-driven decision-making [3]. Studies have reported that CGM use is also associated with improved diabetes-related quality of life, including reduced anxiety related to glucose fluctuations, fewer finger-prick tests, and increased confidence in self-management, as well as a reduced frequency and duration of hypoglycaemic episodes, particularly for individuals on insulin therapy [4,5]. As a result, CGM technology has been widely integrated into standard diabetes care in many high-income countries, particularly for individuals with type 1 diabetes (T1D) and those on intensive insulin therapy [6,7]. In countries such as the United States, the United Kingdom, and Germany, CGMs are routinely used in clinical practice and often reimbursed by national health systems or insurance providers [8]. Their integration has contributed to improved glycaemic outcomes, including reductions in HbA1c, increased time-in-range, and fewer diabetes-related events, and has become a key component of personalised diabetes management in these settings [5].

Despite the proven benefits of CGM, its adoption in low- and middle-income countries (LMIC) remains low. High costs, lack of insurance coverage, limited healthcare infrastructure, and shortages of trained HCPs hinder widespread adoption and sustained use. Additionally, competing health priorities and limited awareness among both clinicians and patients about the potential of CGM further constrain its integration into routine diabetes care in these settings.

In South Africa, access to CGMs remains largely restricted to the private sector, with little evidence of their use in the public health sector. A few studies have explored CGM use among young adults, pregnant women, and HCPs in both the public and private sectors [9–12]. However, these studies are limited by small sample sizes and short follow-up periods. Understanding how CGM can be adopted, accessed, and sustainably implemented in low-resourced settings is critical for promoting health equity, addressing the growing burden of diabetes, and improving outcomes in resource-constrained settings. Identifying the facilitators and barriers to CGM introduction in resource-limited settings is also crucial for designing context-appropriate strategies that support effective and equitable implementation. These insights can inform policy and practice, guide resource allocation, and ensure that CGM technologies are accessible, acceptable, and sustainable within local healthcare systems.

This study aimed to assess the feasibility and acceptability of CGM use among participants enrolled in the first three-arm pragmatic randomised controlled trial on CGM in South Africa, which included people living with T1D, as well as their caregivers and HCPs involved in the study implementation. The objectives of this qualitative study were two-fold: (i) to explore the perceptions and experiences of CGM use and (ii) to identify key barriers and facilitators to CGM implementation in the public healthcare sector in South Africa.

## Methods

### Study design

This qualitative study was embedded within a larger randomised control trial; Access to CGMs for Equity in Diabetes Management (ACCEDE) study, involving people living with T1D who receive diabetes care in the public health sector in South Africa [13]. Data collection was conducted through FGDs and SSIs. These approaches facilitated a comprehensive exploration of participants’ experiences in the ACCEDE study, providing insights into their personal experiences with diabetes management and CGM use. The discussion guides were developed collaboratively by the study team, including implementation scientists, clinicians, and social scientists, and were informed by the study objectives, existing literature, and the socioecological framework. Data collection was conducted by two trained social scientists with formal training and experience in qualitative research methods, including facilitation of focus group discussions (FGDs) and semi-structured interviews (SSIs). Four main thematic areas were explored during data collection using guiding questions and appropriate probes to understand (i) knowledge and perceptions around diabetes management and CGMs, (ii) lived experiences of diabetes self-management using CGMs, (iii) facilitators and barriers to CGM use or self-monitoring methods and (iv) relationships with HCPs/patients: for people living with T1D and their caregivers, we explored their relationships with HCPs, while for HCPs, we focused on their relationships with patients and caregivers. Participants were encouraged to highlight any other relevant topics for discussion (see discussion guides in S1 File).

### Study sample

Participants were recruited from the larger ACCEDE study cohort, which was a three-arm pragmatic randomised controlled trial on CGM use among people living with T1D in South Africa. The study sites were specialised diabetes clinics situated in three large, tertiary-level hospitals; two in Cape Town (Groote Schuur Hospital and Red Cross War Memorial Children’s Hospital) and one in Pretoria (Steve Biko Academic Hospital). Details of the pragmatic randomised trial are described elsewhere [13]. Briefly, participants in the trial were randomly assigned to Arm 1 (continuous CGM use), Arm 2 (periodic CGM use – i.e., one CGM every 3 months), or Arm 3 (standard of care – i.e., self-monitoring of blood glucose (SMBG) and no CGM use) for an intervention period of 9 months and a total of 15 months’ follow-up. Trial participants who had completed at least three scheduled trial visits were eligible to participate in this qualitative study, as this ensured they had sufficient exposure to the study intervention (i.e., CGM use) and could therefore provide informed and meaningful insights based on their experiences. Recruitment was conducted on a rolling basis between July 2024 and February 2025 across the three study sites. Participants randomised to the standard-of-care arm (Arm 3) were included to explore hypothetical perceptions of CGM use, perceived barriers, and social acceptability in the absence of direct exposure. This group represents the majority of public-sector patients who would initially encounter CGM as a new technology.

Recruitment of participants took place from 1^st^ July 2024 until 7^th^ August 2024 in Pretoria and from 18^th^ November 2024 until 5^th^ February 2025 in Cape Town. A pragmatic convenience sampling approach was used to recruit eligible participants (i.e., people living with T1D and caregivers) during scheduled trial visits or contacted telephonically by trained research assistants. Efforts were made to ensure representation across participant categories (adults, adolescents, caregivers) and trial arms (continuous CGM, intermittent CGM, standard of care), across study sites. This allowed for the inclusion of participants with varying ages, sex, and experiences of CGM exposure. Not all eligible participants were included in the qualitative study. Participation was voluntary, and some individuals declined due to time constraints, work or school commitments, or transport challenges. Where possible, reasons for declining to participate in the FGDs were documented. All HCPs involved in the diabetes management of ACCEDE trial participants at the study sites were eligible to participate in SSIs.

### Data collection

Data collection was carried out in two distinct phases: between July and August 2024 in Pretoria and from November 2024 to February 2025 in Cape Town by two independent social scientists. M.S and A.D are both South African, female, public health researchers with formal training (PhD-level) in qualitative research and experience in public health studies. Neither facilitator was involved in the clinical care of participants. To minimise power imbalances, facilitators emphasised their independent research role (no previous relationship with participants prior to study commencement), encouraged open dialogue, and assured participants that responses would not affect their care. The FGDs were conducted in a private room on the hospital premises, ensuring a familiar and safe environment for participants. Caregivers were asked to leave adolescent FGDs to promote honest discussion. Interviews with HCPs were scheduled according to participants’ availability to minimise disruption to their clinical duties and were conducted in their offices to maintain confidentiality and privacy. FGDs were conducted in English, but participants could speak in their preferred local language. The facilitator, who spoke at least one of the commonly spoken languages, provided translation as needed. Fieldnotes were taken during the FGDs/SSIs. Each SSI lasted between 20–60 minutes, while FGDs lasted between 45 minutes and 3 hours, depending on participants’ responsiveness and group dynamics. The FGDs were stratified by randomisation arm and participant type: adults (18 years and older), adolescents (14–17 years), and caregivers of minor participants, to facilitate open dialogue and ensure that the perspectives of each sub-group were fully explored. Due to the sample size and randomisation allocation, within the main study, only one FGD per participant type was feasible to be conducted at each study site with no repeat interviews. The qualitative sample size was guided by pragmatic constraints of the embedded trial design and the goal of achieving thematic sufficiency through concurrent data collection and analysis of all FGD and SSI data and weekly team meetings to review emerging themes. Thematic sufficiency was reached when no substantially new themes or insights emerged during iterative analysis across participant groups and sites.

Both FGDs and SSIs were audio-recorded and transcribed verbatim by a trained, multi-lingual transcriber to ensure accuracy and preserve the cultural significance of transcripts. The social scientists reviewed all transcripts for completeness and accuracy. The transcripts were not returned to participants, nor did they provide feedback. Two other female implementation science researchers (L.M. and E.M.C.) met weekly to discuss emerging themes from the interviews and FGDs.

### Ethical considerations

Ethics approval for the study was obtained from the University of Cape Town (Ref: 558/223) and the University of Pretoria (Ref: 330/2023). Additional approvals were obtained from the provincial health departments (NHRD Ref: GP_202307_092 and WC_202307_119858), where the study was conducted. Written informed consent was obtained from all participants. Minor participants provided written assent, and parental informed consent was obtained from at least one parent or legal guardian for all participants under 18 years of age, in accordance with local ethics committee requirements.

### Data management and analysis

Consistent with previous conceptualisations [14], feasibility was defined as the extent to which CGMs could be practically implemented within the public healthcare context, including considerations of training, infrastructure, affordability, and system readiness, consistent with previous conceptualisations of feasibility, while acceptability was defined as participants’ cognitive and emotional responses to CGM use, including perceived usefulness, comfort, convenience, and social implications. During analysis, data from participants with direct CGM experience (Arms 1 and 2) were analytically distinguished from data generated by participants without CGM exposure (Arm 3). Findings from Arm 3 were interpreted as anticipated perceptions and were not used to infer experiential feasibility or acceptability.

Thematic content analysis following Braun and Clarke’s six-step process was used to analyse both the FGDs and SSIs [15]. The six steps include: 1) familiarising oneself with the data, 2) generating initial codes, 3) searching for themes, 4) reviewing the themes, 5) defining and naming the themes, and 6) producing the report [13]. Two independent researchers with training in qualitative and implementation science coded the transcripts using MAXQDA 2022 (VERBI Software, Berlin, Germany). A combined deductive–inductive approach was employed: an initial coding framework was informed by the study objectives and the socioecological model, while additional codes and themes were generated inductively from the data, enabling the findings to emerge organically rather than being shaped by pre-existing concepts (see coding tree in S2 File). The analysis team met weekly to review and refine the coding structure, resolve any coding discrepancies through consensus, resulting in a comprehensive and well-defined coding framework. Reflexive discussions were used to explore alternative interpretations and ensure analytic consistency. Rather than calculating formal inter-coder reliability metrics, rigor was maintained through iterative comparison of codes, reflexive team dialogue, and transparent documentation of analytic decisions.

### Socioecological model

The Socioecological Model (SEM) provides a useful framework for understanding how individual, interpersonal, community, and societal factors influence behaviours or outcomes [16]. By emphasising the dynamic interplay between these levels, the SEM supports a comprehensive approach to analysing health outcomes and evaluating the effectiveness of interventions. In this study, the SEM was applied to interpret findings and identify key facilitators and barriers to the introduction of CGMs in South Africa’s public health sector.

We followed the COREQ 32-item checklist to guide study design, conduct, analysis, and reporting [17]. (see COREQ checklist in S3 File). Findings are presented under feasibility and acceptability to distinguish between practical implementation considerations and participants’ perceptions and experiences of CGM use.

## Results

A total of 15 FGDs and 16 SSI were conducted with 107 participants, categorised as people living with T1D (n = 75), caregivers (n = 17), and HCPs (n = 15), who were included in the study. Table 1 shows the breakdown of the number of FGDs and SSIs by participants and study sites.

**Table 1:** Summary of data collection by method, type of participant, and site.

Data Collection Method	Arm	Sample	Study Site 01	Study Site 02	Study Site 03
1 FGD	Arm 1	Adults	5	12	NA
1 FGD	Arm 2	Adults	4	9	NA
1 FGD	Arm 3	Adults	4	9	NA
1 FGD	Arm 1	Adolescents	5	NA	6
1 FGD	Arm 2	Adolescents	4	NA	5
1 FGD	Arm 3	Adolescents	3	NA	10
1 FGD	Arm 1	Caregivers	NA	NA	7
1 FGD	Arm 2	Caregivers	NA	NA	8
1 FGD	Arm 3	Caregivers	NA	NA	2
16 SSI	NA	HCPs	5	6	5

NA: Not applicable

Study Site 01: Steve Biko Academic Hospital (combined paediatric/adolescent and adult diabetes clinics; Study Site 02: Groote Schuur Hospital – Diabetes Centre (adult diabetes clinic); Study Site 03: Red Cross War Memorial Children’s Hospital (paediatric and adolescent diabetes clinic).

At Site 01, 50/109 participants enrolled in the parent trial were eligible for the qualitative study at the time recruitment concluded. At Sites 02 and 03, all participants enrolled in the parent trial were eligible and were approached to participate (n = 77 and n = 61, respectively). Participation rate in FGDs at Site 01 was 25/50; Site 02; 30/77; and Site 03; 38/61. Where possible, reasons for declining to participate in the FGDs were documented.

### Participant characteristics

Table 2 summarises participants’ socio-demographic characteristics. Gender was not independently assessed; sex was recorded as a biological variable. Healthcare providers included endocrinologists (including paediatric endocrinologists), diabetologists, medical doctors, and diabetes specialist nurses/nurse educators who had completed a university degree or higher.

**Table 2:** Sociodemographic characteristics of study participants (N = 107).

Variable	PLWD	Caregivers	HCPs
Age (years)			
14-17	32/75 (43%)	0	0
18-29	21/75 (28%)	2/17 (12%)	0
30-49	20/75 (27%)	13/17 (76%)	7/15 (47%)
50-68	2/75 (3%)	2/17 (12%)	8/15 (53%)
Sex			
Male	25/75 (33%)	2/17 (12%)	4/15 (27%)
Female	50/75 (67%)	15/17 (88%)	11/15 (73%)
Highest level of education completed			
University Degree or Higher	7/75(9%)	5/17 (29%)	15/15 (100%)
Vocational/College	3/75 (4%)	1/17 (6%)	0
Secondary School	33/75 (44%)	7/17 (41%)	0
Primary School	30/75 (40%)	4/17 (24%)	0
None (i.e., primary school not completed)	2/75 (3%)	0	0
Employment status			
Employed	15/75 (20%)	10/17 (59%)	15/15 (100%)
Unemployed	25/75 (33%)	7/17 (41%)	0
Student	35/75 (47%)	0	0

**Fig 1:**
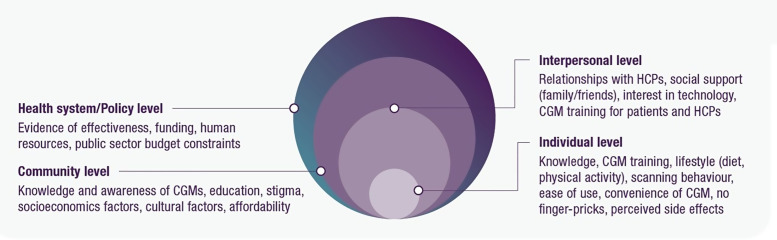
Factors associated with the feasibility and acceptability of CGMs from a patient and HCP perspective according to the socioecological model (SEM).

Thematic analysis of FGDs and SSIs revealed a range of factors influencing the feasibility and acceptability of CGM use. These are presented according to the Socioecological Model (Fig 1), beginning at the individual level and expanding through interpersonal, community, and health system domains. Although data collection occurred at different time points across Pretoria and Cape Town, no major site-specific or time-related variations in core themes were observed, suggesting consistency in participant experiences despite differing clinic contexts and stages of CGM familiarity.

(i) **Individual-level factors**

**Feasibility:** *Ease of use*

Participants highlighted the ease of use and immediate feedback provided by the CGM, which allowed them to manage their glucose levels more proactively:

*“It’s so easy, it gives you better control. So, you can test it every two hours and you see, okay, this is now 8.3, calm down now, drink a little water. So, the scanner is amazing.” (FGD, Arm 2, Adult participant)*

Some adolescents also found CGM more convenient than traditional methods, noting that their caregivers could scan at night to detect lows they could not feel themselves, providing them with some reassurance:

*“I think it’s much easier. Even my mom also thinks that it’s much easier. Especially at night because if it’s low at night I can’t feel it, I can’t hear myself, that’s very dangerous. So, like she just scans, it’s like more convenient for her.” (FGD, Arm 2, Adolescent)*

While the CGM device was reportedly easy to use, HCPs reported that there were some technical challenges, such as sensor insertion and the CGM not remaining on for the duration of the 14 days. Although the challenges were minimal, they did require attention, especially during the early stages of the sensor insertion.

*“In the beginning, there was like a little bit of difficulty with insertion, so they were struggling...but on the whole, no, I think everyone eventually very quickly caught on how to use it and how to insert it and how to keep it in place.” (HCP, 38, Female)*

*“The fact that it falls off and it doesn’t complete the 14-day cycle... there are times when the reader doesn’t want to pick up the reading.” (HCP, 56, Female)*

HCPs generally viewed CGM implementation positively, recognising its potential to enhance diabetes management. They identified several challenges that could affect feasibility, including the need for comprehensive training, dedicated diabetes nurse educators, technical troubleshooting support from the manufacturer, and sustainable funding.

HCPs emphasised that adequate human resources and multidisciplinary teamwork, alongside thorough training for both healthcare staff and patients, are essential for the successful integration of CGMs into clinical practice.

**Feasibility:** *Importance of CGM training for HCPs*

*“Healthcare workers would need some training as to what this does, how it works, and what the goals are and how to achieve them. I think that’s relatively simple...the rest is how do we support a multidisciplinary team to support the patient in using this.” (HCP, 62, Male)*

*“It doesn’t help to give patients access to CGM, but no one knows how to read or download the report, right? So, you’d have to have some kind of technological support in terms of that. So, you’ll have to have a computer, you’d have to have a doctor with experience in CGM or someone who can read the report, who can give advice, adequate advice, otherwise that’s also not appropriate.” (HCP, 34, Female)*

**Acceptability:** *Convenience of CGM*

The convenience of the CGM was reported by people living with T1D as a key factor for acceptability. Many people living with T1D expressed that the CGM was far easier to use than the traditional glucose meter. Participants also reported that they had become increasingly dependent on the device and preferred using the CGM over the SMBG.

*“I call it a glucometer on the go…It’s like it’s always available for me.” (FGD, Arm 1, Adult participant)*

*“I think the machine helps. It really helps. Because it helps with anxiety. Also, to continuously check it so I’m not worried all the time. I think it would have been worse with the finger-prick, suddenly you see big numbers now.” (FGD, Arm 1, Adolescent participant)*

**Acceptability:** *Discreet monitoring*

Participants also highlighted the discreet nature of the CGM as a key advantage, particularly when compared to traditional finger-prick glucose meters. For many, the ability to check glucose levels quickly and quietly in public settings reduced the sense of stigma and self-consciousness often associated with diabetes management.

*“With the CGM, I can just take my (reader) and scan. It’s quick and doesn’t attract attention, unlike the glucometer.” (FGD, Arm 2, Adult Participant)*

*“I can even put it anywhere where people can’t see it, and now I’m feeling like it makes life very much easier.” (FGD, Arm 1, Adult Participant)*

**Acceptability:** *Not having to finger-prick*

The reduction in the physical discomfort associated with finger pricking was another significant factor that increased the acceptability of the CGM. Participants reported that the CGM allowed them to monitor their glucose levels without the burden and discomfort of pricking their fingers.

*“I hate pricking my fingers. It’s so sensitive. When I prick my finger, it still hurts, because my skin is very soft. I have very sensitive skin, so at least with the sensor I don’t have to prick.” (FGD, Arm 1, Adult participant)*

*“No more pricking. Like there’s no more pain, you know, it’s better because my fingertips even those black dots that the pricker leaves, they’re not there anymore.” (FGD, Arm 2, Adolescent participant)*

These perspectives were echoed by HCPs, who also recognised the advantages of CGM in reducing the physical and emotional burden of traditional blood glucose monitoring. They noted that the pain and discomfort associated with frequent finger-pricking often deterred adolescents from regular monitoring, contributing to poor glycaemic control.

*“The teenagers don’t want to prick, because it’s painful, at least with the scanner it’s simple and painless” (HCP, 38, Female)*

*“Well, I think it definitely contributes to the comfort of the child that you’re excluding multiple fingerprints a day.” – HCP (Diabetes Nurse Educator)*

**Acceptability:** *Improved patient knowledge of diabetes*

HCPs highlighted that CGMs had enhanced patient understanding of their condition. By providing continuous feedback, patients have been able to see how different foods, activities, and medications affect their blood glucose levels. This real-time insight has enabled them to make more informed decisions.

*“Many patients now realise that diabetes management goes beyond just finger pricks and insulin injections. They’ve started to understand the importance of their medication, and when they follow advice, they see real improvements. The support they receive also plays a crucial role, giving them confidence and better results.” (HCP, 56, Female)*

Participants who had used a CGM were reportedly scanning between 4–8 times a day and checking their glucose levels more often than when they were on the glucose meter.

*“I’m scanning much more than I used to on the glucometer. When I was using the glucometer, I was testing maybe two times a day, or sometimes never. Now I can test even 10 times a day, or just when I feel like it, if I feel like something is off in my body, I take out my scanner and I test.” (FGD, Arm 1, Adult)*

On the other hand, people living with T1D shared a common downside to frequent scanning, which was the fear of seeing high glucose readings, which made them feel demotivated, leading them to avoid checking their glucose levels altogether in order to prevent disappointment or stress.

*“Ah, sometimes I just don’t want to test because it’s really not nice seeing those high numbers! I just wait until later and maybe it will go down, because no matter what I do, it’s always high, so it’s better not to check sometimes” (FGD, Arm 1, Adolescent)*

*“When I test, I make sure that it’s going to be a good result. I don’t want high. I make sure I inject before I test because now you see we are drinking tea. So, this tea will raise your sugar (FGD3, Arm 3, Adult).*

**Acceptability:** *Affordability as a significant barrier to CGM access*

However, despite the enthusiasm for CGM technology, several HCPs voiced strong concerns that the high cost of CGMs posed a significant barrier to widespread adoption within the public healthcare system. The issue of affordability was consistently raised as a critical limiting factor, with providers acknowledging that while the clinical and practical benefits of CGMs are clear, financial constraints would likely prevent access for the majority of their patients.

*“I think funding. That’s the only hurdle, really. So, I think everyone else would accept it immediately. All the clinicians would prescribe it if it were available, and most of the patients would choose to use it.” (HCP, 38, Female)*

*“Some of our diabetics don’t even have access to food. They can’t even afford food. So yes, it will benefit them, but priorities are different for different patients. Food accessibility is obviously above a CGM.” (HCP, 34, Female)*

Beyond individual attitudes and behaviours, participants’ experiences with CGMs were strongly shaped by interactions with family members, caregivers, and healthcare providers. These interpersonal dynamics played a pivotal role in supporting, or at times hindering, ongoing use of the technology.

(ii) **Interpersonal level factors**

**Feasibility**

HCPs emphasised that for CGM implementation to be feasible, structured and ongoing training would be essential for both HCPs and patients. This would ensure not only effective use of the device but also continuous patient engagement and sustained impact on diabetes management. As one clinician noted, training and evaluation must go hand-in-hand to support long-term success.

*“We’ll have to make sure that there are training programs both for HCPs as well as for patients. So, I think the two will just have to go together, and there’ll have to be a continuous sort of evaluation of the patient’s involvement in the CGM.”* – HCP (Medical Doctor)

**Acceptability**

HCPs expressed high levels of acceptability overall. Acceptability of CGMs at an interpersonal level was influenced by relationships between people living with T1D and their caregivers and HCPs. CGMs were perceived to provide better insights for HCPs and improved understanding for people living with T1D of how to manage their condition:

**Acceptability:** *Support from HCPs and family members*

Some participants noted that the simplicity of CGM use allowed family members to assist in monitoring, particularly during hypoglycaemic episodes when the patient was incapacitated. This shared use enhanced safety and provided reassurance to both patients and caregivers.

*“Even if my sugar drops to a point where I am unable to do anything, people in my house find it easy to use and check what’s wrong, because it sometimes makes me, like, maybe not able to wake up, you understand? And then they would check me and see that it is low, then they would help me quickly, and then I would be fine. And then eish, without it life becomes difficult, yeah, it is not nice.” (FGD, Arm 2, Adult participant)*

**Acceptability:** *Better insights for HCP*

HCPs highlighted that CGMs offered more comprehensive data compared to traditional self-monitoring of blood glucose. This enhanced data enabled them to better understand individual patient patterns, identify factors contributing to glycaemic variability, and tailor treatment decisions accordingly. Providers also used the CGM data as an educational tool to engage patients in discussions about their behaviours, lifestyle choices, and the impact on glucose control.

*“I get to know the person that I’m speaking to. Because now I can put together their lifestyle, the person, and what their blood glucose control is, why they have a high HbA1c, and why they had a hyperglycaemic event. So, I get to know the patients more in depth.” (HCP, 56, Female)*

*“I mean, when we have the CGM readings, we can sit down with the patient, we can discuss the readings, the profiles that we see, we can discuss what we think influences that profile, what makes the sugar go up and down. And then we can discuss any changes to therapy that we want.” (HCP, 62, Male)*

**Acceptability:** *Distrust/ fear of CGMs*

A few participants raised concerns about data confidentiality and expressed mistrust regarding how the information collected by the devices might be used for purposes beyond diabetes management. However, these concerns were limited to a small subset of participants.

*“Some patients’ husbands were worried that this was like a spyware kind of thing. They weren’t sure what this thing was now all about. ‘Does this do more than just manage my sugar?’ We’re living in an age where everybody’s concerned, you know? So, what is this actually monitoring? So, is it just my sugar, you know?” (HCP, 62, Male)*

Moving beyond personal relationships, broader community and environmental factors, such as school settings, social norms, and peer perceptions, also influence CGM use, particularly among adolescents. These factors contributed to both practical challenges and emotional responses to using the device in public.

(iii) **Community-level factors**

**Feasibility**

Access to healthy food was highlighted as a key factor in the feasibility of CGMs for school-going children with T1D. While CGMs support better glucose monitoring, their impact is limited when children are provided with high-carbohydrate meals at school to supplement their meals. As one diabetes nurse educator explained:

*“Some of the food that they get at school is not good for diabetic children. They are getting food that has lots of carbohydrates. Whereas with type one [* 7 *], they are supposed to eat moderate food, they do need carbohydrates, but to maintain themselves, but not a whole plateful of it.” – HCP (Diabetes Nurse Educator)*

One caregiver highlighted how the visibility of CGM devices can spark curiosity and questions within communities. While this may feel burdensome for some adolescents, it also presents an important opportunity to raise awareness, reduce stigma, and educate others about diabetes and CGM use, ultimately fostering greater community acceptance and support:

*“So, like I mean, it’s summer, he was like, ’I wish I would wear long sleeves every day”, and then I was like, “Why?” I think a lot of people are starting to question what this is. And I don’t think he’s in the mood to explain it.” – Caregiver of Arm 1 participant*

**Acceptability**

Participants randomised to the standard-of-care arm (Arm 3), who had not used CGMs, expressed anticipated concerns regarding device visibility and potential stigma. These perceptions were based on expectations rather than lived experience with the technology. Perceived stigma, particularly in school settings, was highlighted by HCPs as an important consideration for adolescents. Fear of being identified as “*diabetic*” affected how openly participants used the CGM in public, reducing its acceptability in these environments.

**Acceptability:** *Fear of being seen as diabetic and being stigmatised.*

*“You get one or two patients that don’t want to use it because they don’t want to have something on them that people can identify that they have diabetes. That’s predominantly the adolescent group, but that’s the same in state or private.” (HCP, 38, Female)*

*“My biggest concern is it being visible, then I have to always explain to people” (FGD, Arm 3, Adult)*

Despite these reservations, people living with T1D indicated that they would still be interested in using the CGM due to its perceived benefits.

*“So, my thoughts are that this would do me so much good, you know, having the CGM. I don’t mind that people are going to ask questions regardless. I’ve had a series of questions before I can handle the questions.” (FGD, Arm 3, Adults)*

Finally, participants reflected on structural and system-wide influences, including healthcare infrastructure, resource constraints, and national policies. These systemic factors were often perceived as either key enablers or persistent barriers to sustainable CGM integration in the public sector.

(iv) **Health System/Policy level factors**

**Feasibility:** *Benefits of CGM in reducing hospital admissions*

HCPs perceived that CGM use may contribute to fewer hospital admissions, particularly for diabetic ketoacidosis (DKA) and other complications associated with poor glucose control, highlighting that patients who consistently used CGM had fewer episodes of severe hypoglycaemia and hyperglycaemia. However, these observations reflect provider perceptions rather than measured outcomes

*“The admission rate has reduced within the study group. There have definitely been at least two patients who said ‘We were always in the hospital and now, because of the CGM, we haven’t been admitted at all this year’” - HCP (Medical Doctor), 58, Female*

They emphasized that demonstrating the long-term cost-effectiveness of CGMs, particularly in preventing severe diabetes complications, could help secure greater buy-in from health system decision-makers.

*‘The cost-benefit ratio might be better to buy the sensor…because you are going to have to deal with your amputations, theatre time, ICU time, multiple DKA admissions, which require ICU or fluids or treatment in the ward for at least a week. So, from that perspective, if you can do a cost analysis, then maybe you’ll get better buy-in. - HCP (Medical Doctor), 38, Female*

**Feasibility:** *Need for the government to subsidise the cost of CGM*

Most HCPs expressed concerns regarding the high cost of CGMs, highlighting that the devices, while beneficial, were often too expensive for patients. HCPs expressed that despite the long-term benefits of CGM in potentially improving patient health outcomes and reducing complications, the high costs were a significant barrier to broader adoption within the public sector.

*“It’s got to be 100% government funded because if it’s co-payment, you’re still going to have a problem because there are too many people that are not working. So how are they going to pay for it?” (HCP, 56, Female)*

*“The patients can’t afford it. It’s like everything else in the state sector. They look at your income. Most of our patients have zero income; they rely on all the state benefits and stuff. If we want to give it to our patients, they will have to get it for free. They can’t pay for it.” (HCP, 51 Female)*

**Acceptability:** *Critical role of Diabetes Nurse Educators*

The need for continuous support and education was highlighted as critical for optimal use of CGMs. Several HCPs noted that the successful implementation would need more than just the initial training, but would require additional human resources, in particular a diabetes nurse educator to provide ongoing education, training, and psychosocial support to help patients fully benefit from the technology.

*“So, if you’ve got a diabetes educator…if you have constant presence of someone there to reiterate and re-educate them. I think that’s going to make a bigger difference.” (HCP, 38, Female)*

*“I’m the only paediatric endocrinologist here, so sometimes when you have a very busy clinic and then you’ve got a lot of patients to see, you do need extra staff members to help give a better service. So obviously it takes a lot longer when you have CGM. Even though it’s beneficial, you still need to have somebody to download the CGM. If you don’t have anyone to download that, that eats into your time.” (HCP, 38, Female)*

In summary, key facilitators to CGM implementation in the public health sector include the high acceptability of the technology among both patients and providers, its ease of use, perceived improvements in glucose control, and the potential for enhanced patient-provider communication and tailored care. Family and peer support, particularly for adolescents, further encouraged consistent use, while HCPs valued the clinical insights enabled by real-time data. However, several barriers could hinder implementation. Beyond cost and limited affordability, participants described broader structural and contextual barriers. These included transport constraints and food insecurity, which undermined the ability to act on CGM insights; stigma and social visibility of wearing devices, particularly acute among adolescents; and technical difficulties with sensors and apps during the early stages of use. Health system factors were also raised, mainly around the need for provider training and integration of CGM into existing care pathways. Together, these issues highlight that while cost remains a critical concern, a range of social, technical, and systemic barriers must be addressed to ensure sustainable and equitable adoption of CGM in resource-limited settings.

While the findings have been presented according to the SEM levels, some themes, such as stigma, education, and affordability, emerged as crosscutting across the socioecological levels, reinforcing the interdependent nature of factors influencing CGM feasibility and acceptability. Recognising these interconnected influences provides a more holistic understanding of CGM feasibility and acceptability.

Stigma, while most visible at the community level, particularly among adolescents wary of being identified as diabetic, also shaped interpersonal interactions and individual behaviours, leading some participants to avoid wearing or using the device in public.

Education and training needs were echoed across all levels: individuals expressed a desire to better understand their condition and how to use the CGM; HCPs emphasised the need for technical and interpretive training; and at the system level, the absence of diabetes specialist nurses was identified as a key gap in sustaining support. Affordability was a dominant and recurring barrier: at the individual level, many participants lacked income or financial security; at the interpersonal and community levels, limited household resources often competed with other priorities such as food or transport; and at the health system level, HCPs consistently raised concerns about the high cost of CGMs and the lack of government subsidy. These overlapping challenges highlight the importance of a comprehensive, multi-level implementation approach that simultaneously addresses personal, relational, and structural factors to enable sustainable CGM access and use.

## Discussion

This study is the first to explore the acceptability and feasibility of CGM use among people living with T1D, including adolescents, adults, and caregivers of children living with T1D, as well as HCPs, in South Africa’s public healthcare sector. It represents the largest qualitative study on CGM use among people living with T1D in LMICs, contributing important insights for implementation in a resource-limited setting.

### Acceptability and patient empowerment

CGMs were widely viewed as acceptable and valuable, with study participants reporting reduced reliance on finger-pricking, discreet monitoring, and immediate feedback that supported proactive self-management. Many described feeling more in control of their condition and less anxious about glucose fluctuations. It is important to distinguish between experiential acceptability reported by participants with direct CGM exposure and theoretical perceptions expressed by those in the standard-of-care arm. While CGM-experienced participants largely described high acceptability grounded in lived use, concerns raised by non-users primarily reflected expectations about stigma and visibility rather than experience. HCPs echoed the benefits of CGMs, highlighting the clinical utility of real-time glucose data for tailoring treatment decisions. These findings mirror evidence from high-income [18,19] and a limited but growing body of evidence from low- and middle-income settings [9,10,12,14,20,21] and confirm that CGMs can empower patients and enhance provider-patient interactions even in resource-constrained environments. Adolescents participating in a qualitative study in South Africa reported enhanced autonomy in diabetes self-management and increased feelings of empowerment, which were associated with improved perceptions of social belonging and quality of life [12]. Similarly, a pilot and feasibility study conducted in Kenya and Uganda demonstrated the feasibility of CGM use among young people in East Africa [21].

### Barriers to sustained use

Despite these benefits, barriers at multiple levels were identified. Adolescents in particular reported stigma related to visible devices and fears of being identified as “*diabetic*”, consistent with findings in South Africa, Malawi and Pakistan [20,22]. Some participants expressed distress from frequent glucose checks, leading to avoidance behaviours, highlighting that the acceptability of CGMs cannot be considered uniformly across all groups. To improve adoption, implementation strategies in public healthcare settings must address data privacy concerns, reduce stigma through community awareness campaigns, and provide education and psychosocial support, particularly for adolescents. At the structural level, food insecurity and unemployment limited the capacity to respond to CGM data, while HCPs pointed to persistent shortages of trained staff. Most critically, the high cost of devices was perceived as the greatest obstacle to equitable access in the public sector, with both patients and providers noting that without government support, uptake would remain limited.

### Importance of education and support

A dominant theme was the need for sustained training and psychosocial support. Consistent with findings from qualitative studies in high-income countries [23,24], participants emphasised that access to CGMs alone was insufficient without the skills to interpret data and adjust management accordingly. HCPs highlighted the importance of multidisciplinary teams, especially the inclusion of diabetes specialist nurses to provide structured education and ongoing follow-up. This reflects broader evidence that successful integration of new health technologies requires supportive systems and workforce strengthening, not only device provision.

While participants reported improved awareness of glycaemic patterns and greater perceived control over self-management, some also described anxiety and avoidance in response to high readings. These findings are not contradictory but reflect the dual effects of intensified self-monitoring, whereby real-time feedback may support behavioural regulation while simultaneously increasing emotional burden in the absence of adequate psychosocial support.

### Policy and health system implications

The findings indicate that CGM introduction in South Africa’s public health sector is both feasible and acceptable, but its success hinges on policy-level action. Investment is needed to subsidise or fully fund CGMs for public sector patients, alongside implementation strategies to reduce stigma and strengthen peer and family support. While participants perceived that CGMs could reduce hospital admissions and improve cost-effectiveness, these potential benefits were not empirically assessed in this qualitative study and should be interpreted as stakeholder expectations. Demonstrating the cost-effectiveness of continuous [25,26] or intermittent [27] CGM use, such as reduced hospital admissions for diabetic ketoacidosis, are needed to substantiate these claims and will be critical for securing government buy-in [28]. In addition, expanding the diabetes workforce, particularly diabetes specialist nurses, will be essential to sustain long-term benefits.

### Contribution to the evidence base

This study adds to the sparse evidence on CGM use in LMICs, demonstrating both the promise and the challenges of implementation. CGMs are highly acceptable to patients and providers, with the potential to transform diabetes management. However, effective integration into the public sector requires addressing individual, interpersonal, community, and system-level barriers. Participants and providers perceived current pricing as a barrier to public-sector adoption, suggesting a need for financing mechanisms that do not involve substantial out-of-pocket payments by users to support equitable access. Policy commitments, financing mechanisms, and investment in multidisciplinary support are urgently needed to ensure that CGM technology contributes meaningfully to equitable diabetes care. These findings highlight the importance of structured training, sustained healthcare provider support, and policy-level interventions to improve equitable access to CGM.

The qualitative findings will complement the ACCEDE trial objectives by providing contextual explanations for issues related to equity, adherence, acceptability, and feasibility. For example, high acceptability and perceived convenience help explain sustained engagement with CGM among participants in the intervention. At the same time, barriers such as food insecurity, transport costs, and stigma highlight structural factors that may limit equitable access and consistent CGM use. Healthcare provider concerns regarding affordability and staffing constraints further contextualise the health system challenges that influence scalability within the public healthcare sector.

While the findings are context-specific, they are likely transferable to similar resource-limited settings where access to diabetes technologies remains a challenge. Further research should focus on implementation pilots to test scalable service delivery models for CGM within South Africa’s public healthcare system, including task-shifting approaches and integration with diabetes nurse educator support. Quantitative evaluations of clinical outcomes and cost-effectiveness are needed to substantiate stakeholder perceptions and inform policy decisions. Additionally, community-based interventions aimed at reducing stigma, increasing awareness and education, and strengthening adolescent support systems may enhance sustained uptake.

### Limitations of the study

This study had some limitations. First, the qualitative sampling was pragmatically shaped by the structure and randomisation of the main trial, which limited the number of FGDs per participant category at each site. While this may have constrained the depth of within-group exploration, thematic consistency across sites and participant groups supports the credibility of the findings. As we did not formally assess saturation within each participant group, we report on thematic sufficiency. Secondly, some eligible participants were unable to attend FGDs due to scheduling conflicts with work or school, or limited access to transportation, despite prior communication that transport costs and time would be reimbursed. While flexible scheduling was attempted, it did not consistently improve attendance. Nevertheless, the discussions yielded rich and meaningful data due to the diversity in the study sample. Thirdly, conducting FGDs at the hospital where participants receive care may have limited open expression due to concerns about potential repercussions. However, this setting offered familiarity and accessibility, and sessions were facilitated by an independent, multilingual researcher to reduce perceived bias. Facilitators employed trust-building techniques to foster a safe and open environment, and caregivers of adolescent participants were asked to leave the room to ensure participant comfort and privacy. Finally, this study was conducted in tertiary-level hospitals, which may limit transferability to primary care or rural settings.

## Conclusion

Our findings demonstrate that CGMs are both acceptable and feasible within South Africa’s public health sector, as reported by people living with T1D, their caregivers, and HCPs. Successful integration into routine diabetes care will require more than device availability alone. A multi-level response is needed, one that addresses individual motivation and support, strengthens interpersonal care relationships, reduces community-level stigma, and ensures health system readiness. Crucially, sustained investment in provider training, structured education, and national policies that address affordability and access will be essential to bridge the gap between feasibility and equitable implementation. We believe these findings offer a valuable perspective for policy and programme efforts that aim to make CGMs more accessible, equitable, and part of routine care.

## Supporting information

S1 File Focus group and semi-structured interview guides developed to explore knowledge, perceptions, experiences, and implementation considerations related to continuous glucose monitoring. (PDF)

S2 File Coding tree demonstrating how initial deductive codes and inductively generated codes were organised into themes and sub-themes across individual, interpersonal, community, and health system levels. (PDF)

S3 File Completed COREQ (Consolidated Criteria for Reporting Qualitative Research) Checklist for Interviews and Focus Groups. (PDF)
