# Supplementary material for: Exploring the feasibility and acceptability of Continuous Glucose Monitoring among people with type 1 diabetes and healthcare providers in South Africa’s public sector: A qualitative study
Source: PLoS One. 2026 Jul 16;21(7):e0352590. doi: 10.1371/journal.pone.0352590 (PMC13374883; doi:10.1371/journal.pone.0352590)
Supplement: S1 File — (PDF) [file pone.0352590.s001.pdf]

STUDY TITLE: Three-arm pragmatic randomized study on the effectiveness, feasibility, acceptability, and cost of the use of continuous glucose monitoring devices among people living with type 1 diabetes in South Africa

---

Focus Group Discussion (FGD) Guide: Adults (Arm 1)

Theme 1: Knowledge and perceptions around diabetes management and CGMs:

1. What comes to your mind when you hear the word **diabetes management or diabetes control**?

2. What was your **understanding of CGMs before your participation** in this study? [Probe: Did you know about CGMs before the study? Source of information?]

3. What were your **initial thoughts/feelings** about using CGMs? [Probe]: Excited, curious, unsure…

4. What is your **current understanding of CGM** and their use in diabetes management?

Theme 2: Lived experiences of diabetes self-management using CGMs:

5. How often do you **wear your CGM device**? [Probe]: What factors affect how often you wear your CGM device?

6. How often do you **scan your CGM device (per day, per week, etc.)?** [Probe]: What factors affect how often you scan your CGM device?

7. How has **using a CGM device affected your diabetes management and glucose control?** [Probe]: What impact has the CGM had on your diabetes management/how you manage your diabetes?

8. How has **using a CGM device affected your daily routine/your life?** [Probe]: What impact has the CGM had on your life?

9. What **changes** (if any) have you noticed in your **quality of life since using a CGM** device?

10. How do you feel while using a **CGM?** [Probe]: How do you feel about wearing a CGM/scanning?

11. What do people in your life (i.e. your community, family, friends, partner) think about your CGM use?

    a. ONLY if directly prompted by participants: Have you ever felt **embarrassed or self-conscious about using your CGM device**? How do you deal with those feelings?

Theme 3: Facilitators and Barriers to CGM use/SMBG:

12. What **challenges** (if any) have you experienced **with using your CGM** device? How did you address them?

13. Which factors (if any) **motivate** you to use/continue to use a CGM device?

14. How do you feel about the **results/readings from your CGM device?**
    [Probe]: Do you feel confident or trust the readings you get from your CGM device?

15. What **challenges** (if any) have you experienced **with using your SMBG** device? How did you address them?

16. Which factors (if any) motivate you to use/continue to use an SMBG device?

17. How do you feel about the **results/readings from your SMBG device?**
    [Probe]: Do you feel confident or trust the readings you get from your SMBG device?

18. What factors would influence your decision to use/not use a CGM (even after the study has ended)?

Theme 4: Relationships with HCPs:

19. How would you describe your **relationship with your doctor/nurse** with respect to diabetes management?

20. How has using a **CGM device affected your relationship with your doctor/nurse?**
    [Probe]: Have you noticed any changes in how you communicate with your doctor/nurse?

<u>Closing statement:</u>

Before closing the session, is there **anything else you would like to add that is important to consider that we did not cover in this discussion?**

We have come to the end of the discussion. Thank you again for your time, feedback and participation. Your input is very important for us and will be valuable for future considerations regarding diabetes self-management. Please do not hesitate to contact me if you have any other questions about the study or our discussion today.

[END RECORDING]

Focus Group Discussion (FGD) Guide: Adults (Arm 2)

Theme 1: Knowledge and perceptions around diabetes management and CGMs:

1. What comes to your mind when you hear the word **diabetes management or diabetes control**?

2. What was your **understanding of CGMs before your participation** in this study? [Probe: Did you know about CGMs before the study? Source of information?]

3. What were your **initial thoughts/feelings** about using CGMs? [Probe]: Excited, curious, unsure…

4. What is your **current understanding of CGMs** and their use in diabetes management?

Theme 2: Lived experiences of diabetes self-management using CGMs:

5. How often do you **wear your CGM device**? [Probe]: What factors affect how often you wear your CGM device?

6. How often do you **scan your CGM device (per day, per week, etc.)?** [Probe]: What factors affect how often you scan your CGM device?

7. How has **using a CGM device affected your diabetes management and glucose control?** [Probe]: What impact has the CGM had on your diabetes management/how you manage your diabetes?

8. How has **using a CGM device affected your daily routine/your life?** [Probe]: What impact has the CGM had on your life?

9. What **changes** (if any) have you noticed in your **quality of life since using a CGM** device?

10. How do you feel while using a **CGM?** [Probe]: How do you feel about wearing a CGM/scanning?

11. What do people in your life (i.e. your community, family, friends, partner) think about your CGM use?
    a. ONLY if directly prompted by participants: Have you ever felt **embarrassed or self-conscious about using your CGM device**? How do you deal with those feelings?

Theme 3: Facilitators and Barriers to CGM use/SMBG:

12. What **challenges** (if any) have you experienced **with using your CGM** device? How did you address them?

13. Which factors (if any) **motivate** you to use/continue to use a CGM device?

14. How do you feel about the **results/readings from your CGM device?**
[Probe]: Do you feel confident or trust the readings you get from your CGM device?

15. What **challenges** (if any) have you experienced **with using your SMBG** device? How did you address them?

16. Which factors (if any) motivate you to use/continue to use an SMBG device?

17. How do you feel about the **results/readings from your SMBG device?**
[Probe]: Do you feel confident or trust the readings you get from your SMBG device?

18. What factors would influence your decision to use/not use a CGM (even after the study has ended)?

Theme 4: Relationships with HCPs:

19. How would you describe your **relationship with your doctor/nurse** with respect to diabetes management?

20. How has using a **CGM device affected your relationship with your doctor/nurse?**
[Probe]: Have you noticed any changes in how you communicate with your doctor/nurse?

<u>Closing statement:</u>

Before closing the session, is there **anything else you would like to add that is important to consider that we did not cover in this discussion?**

We have come to the end of the discussion. Thank you again for your time, feedback and participation. Your input is very important for us and will be valuable for future considerations regarding diabetes self-management. Please do not hesitate to contact me if you have any other questions about the study or our discussion today.

[END RECORDING]

Focus Group Discussion (FGD) Guide: Adults (Arm 3)

Theme 1: Knowledge and perceptions around diabetes management and CGMs:

1. What comes to your mind when you hear the word **diabetes management or diabetes control**?

2. What was your **understanding of CGMs before your participation** in this study? [Probe: Did you know about CGMs before the study? Source of information?]

3. What were your **initial thoughts/feelings** about using CGMs? [Probe]: Excited, curious, unsure…

4. What is your **current understanding of CGMs** and their use in diabetes management?

Theme 2: Lived experiences of diabetes self-management using a self-monitoring blood glucose (SMBG) device (i.e. a glucometer):

5. How often do you **check your blood glucose using a glucometer/Accuchek**? [Probe]: What factors affect how often you check/test your blood glucose?

6. How has **using a glucometer affected your diabetes management and glucose control?**

7. How has **using a glucometer affected your daily routine/your life?** [Probe]: What impact has the CGM had on your life?

8. What do people in your life (i.e. your **community, family, friends, partner**) think about you using a glucometer?
    a. ONLY if directly prompted by participants: Have you ever felt **embarrassed or self-conscious about using your glucometer**? How do you deal with those feelings?

Theme 3: Facilitators and Barriers to SMBG:

9. What **challenges** (if any) have you experienced **with using your glucometer**? How did you address them?

10. Which factors (if any) **motivate** you to use/continue to use your glucometer?

11. How do you feel about the **results/readings from your glucometer?** [Probe]: Do you feel confident or trust the readings you get from your glucometer?

12. What factors would influence your **decision to use/not use a CGM** (even after the study has ended)?

Theme 4: Relationships with HCPs:

13. How would you describe your **relationship with your doctor/nurse** with respect to diabetes management?

14. How has using **a SMBG device/glucometer** affected your relationship with your healthcare provider or care team? Have you noticed any changes in how you communicate with your doctor/nurse?

Closing statement:

Before closing the session, is there **anything else you would like to add that is important to consider that we did not cover in this discussion?**

We have come to the end of the discussion. Thank you again for your time, feedback and participation. Your input is very important for us and will be valuable for future considerations regarding diabetes self-management. Please do not hesitate to contact me if you have any other questions about the study or our discussion today.

[END RECORDING]

Focus Group Discussion (FGD) Guide: Adolescents (Arm 1)

Theme 1: Knowledge and perceptions around diabetes management and CGMs:

21. What comes to your mind when you hear the word **diabetes management or diabetes control**?

22. What **did you know about CGMs before** the study?
    [Probe: Did you know about CGMs before the study? Source of information?]

23. What were your **initial thoughts/feelings** about using CGMs?
    [Probe]: Excited, curious, unsure…

24. What is your **current understanding of CGM** and their use in diabetes management?

Theme 2: Lived experiences of diabetes self-management using CGMs:

25. What do you think about your CGM? Do you **like using it or not,** and why?
    [Probe: What do you like the most about it? And the least?]
26. How often do you **wear your CGM device**?
    [Probe]: What factors affect how often you wear your CGM device?

27. How often do you **scan your CGM device (per day, per week, etc.)?**
    [Probe]: What factors affect how often you scan your CGM device?

28. How has **using a CGM device affected your diabetes management and glucose control?**
    [Probe]: What impact has the CGM had on your diabetes management/how you manage your diabetes?

29. How has **using a CGM device affected your daily routine/your life?**
    [Probe]: What impact has the CGM had on your life?

30. What **changes** (if any) have you noticed in your **quality of life since using a CGM** device?

31. What do **people in your life** (i.e. your school, community, family, friends, partner) think about your CGM use?
    [Probe]: Have you told anybody at school/friends about your condition?
32. Have you ever felt **embarrassed about using your CGM device**? If so, why? How do you deal with those feelings?

Theme 3: Facilitators and Barriers to CGM use/SMBG:

33. What **features of your CGM do you find most helpful**? Is there anything you wish it could do that it currently does not? Why?
    a. Can you give us some specific examples?
34. What **issues or problems** (if any) have you experienced **with using your CGM** device? How did you handle them?

35. Which factors (if any) **motivate** you to use/continue to use a CGM device?

36. How do you feel about the **results/readings from your CGM device?**
    [Probe]: Do you feel confident or trust the readings you get from your CGM device?

37. What **challenges** (if any) have you experienced **with using your SMBG** device (glucometer)? How did you address them?

38. Which factors (if any) **motivate** you to use/continue to use an SMBG device (glucometer)?

39. How do you feel about the **results/readings from your SMBG device** (glucometer)?
    [Probe]: Do you feel confident or trust the readings you get from your SMBG device?

40. What factors would **influence your decision to use/not use a CGM** (even after the study has ended)?

Theme 4: Relationships:

41. Is there anyone else besides yourself, **who is involved in your diabetes management (in general)**?
    [Probe]: Such as, parents, siblings, teachers, partners? What is their role?

42. Is there anyone else besides yourself, **who is involved in your diabetes management using a CGM**?
    [Probe]: Such as, parents, siblings, teachers, partners? What is their role?

43. How do you feel about **sharing your CGM data** with others, such as your parents or doctors?

44. How would you describe your **relationship with your doctor/nurse** with respect to diabetes management?

45. How has using a **CGM device affected your relationship with your doctor/nurse?**
    [Probe]: Have you noticed any changes in how you communicate with your doctor/nurse?

<u>Closing statement:</u>

Before closing the session, is there **anything else you would like to add that is important to consider that we did not cover in this discussion?**

We have come to the end of the discussion. Thank you again for your time, feedback and participation. Your input is very important for us and will be valuable for future considerations regarding diabetes self-management. Please do not hesitate to contact me if you have any other questions about the study or our discussion today.

[END RECORDING]

Focus Group Discussion (FGD) Guide: Adolescents (Arm 2)

Theme 1: Knowledge and perceptions around diabetes management and CGMs:

1. What comes to your mind when you hear the word **diabetes management or diabetes control**?

2. What **did you know about CGMs before** the study?
   [Probe: Did you know about CGMs before the study? Source of information?]

3. What were your **initial thoughts/feelings** about using CGMs?
   [Probe]: Excited, curious, unsure…

4. What is your **current understanding of CGM** and their use in diabetes management?

Theme 2: Lived experiences of diabetes self-management using CGMs:

5. What do you **think about your CGM**? Do you **like using it or not,** and why?
   [Probe: What's what you like the most about it? And the least?]
6. How often do you **wear your CGM device**?
   [Probe]: What factors affect how often you wear your CGM device?

7. How often do you **scan your CGM device (per day, per week, etc.)?**
   [Probe]: What factors affect how often you scan your CGM device?

8. How was your experience **having the CGM on and off during the study?**
   [Probe: How did you feel about having to switch between CGM and your glucometer?]

9. How has **using a CGM device affected your diabetes management and glucose control?**
   [Probe]: What impact has the CGM had on your diabetes management/how you manage your diabetes?

10. How has **using a CGM device affected your daily routine/your life?**
    [Probe]: What impact has the CGM had on your life?

11. What **changes** (if any) have you noticed in your **quality of life since using a CGM** device?

12. How do you feel while using a **CGM?**
    [Probe]: How do you feel about wearing a CGM/scanning?

13. What do **people in your life** (i.e. your school, community, family, friends, partner) think about your CGM use?

a. Have you ever felt **embarrassed about using your CGM device**? Why? How do you deal with those feelings?

Theme 3: Facilitators and Barriers to CGM use/SMBG:

14. What **features of your CGM do you find most helpful**? Is there anything you wish it could do that it currently does not? Why?
    a. Can you give us specific examples?
15. What **issues or problems** (if any) have you experienced **with using your CGM** device? How did you handle them?
16. Which factors (if any) **motivate** you to use/continue to use a CGM device?
17. How do you feel about the **results/readings from your CGM device?**
    [Probe]: Do you feel confident or trust the readings you get from your CGM device?
18. What **challenges** (if any) have you experienced **with using your SMBG** device? How did you address them?
19. Which factors (if any) **motivate** you to use/continue to use an SMBG device?
20. How do you feel about the **results/readings from your SMBG device?**
    [Probe]: Do you feel confident or trust the readings you get from your SMBG device?
21. What factors would **influence your decision to use/not use a CGM** (even after the study has ended)?

Theme 4: Relationships:

22. Is there anyone else besides yourself, **who is involved in your diabetes management using a CGM**?
23. [Probe]: Such as, parents, siblings, teachers, partners? What is their role?
24. How do you feel about **sharing your CGM data** with others, such as your parents or doctors? Does it make you feel uncomfortable or empowered?
25. How would you describe your **relationship with your doctor/nurse** with respect to diabetes management?
26. How has using a **CGM device affected your relationship with your doctor/nurse?**
    [Probe]: Have you noticed any changes in how you communicate with your doctor/nurse?

<u>Closing statement:</u>

Before closing the session, is there **anything else you would like to add that is important to consider that we did not cover in this discussion?**

We have come to the end of the discussion. Thank you again for your time, feedback and participation. Your input is very important for us and will be valuable for future considerations regarding diabetes self-management. Please do not hesitate to contact me if you have any other questions about the study or our discussion today.

[END RECORDING]

Focus Group Discussion (FGD) Guide: Adolescents (Arm 3)

Theme 1: Knowledge and perceptions around diabetes management and CGMs:

1. What comes to your mind when you hear the word **diabetes management or diabetes control**?

2. What **did you know about CGMs before** the study?
   [Probe: Did you know about CGMs before the study? Source of information?]

3. What were your **initial thoughts/feelings** about CGMs?
   [Probe]: Excited, curious, unsure…

4. What is your **current understanding of CGM** and their use in diabetes management?

Theme 2: Lived experiences of diabetes self-management:

5. What do you think about SMBG (glucometer)? Do you **like using it or not,** and why?
   [Probe: What's what you like the most about it? And the least?]
6. How has **using SMBG (glucometer) affected your diabetes management and glucose control?**
   [Probe]: What impact has the SMBG had on your diabetes management/how you manage your diabetes?
7. How has **using a SMBG device affected your daily routine/your life?**
   [Probe]: What impact has SMBG had on your life?
8. What **changes** (if any) have you noticed in your **quality of life since using a SMBG** device?
9. What do **people in your life** (i.e. your school, community, family, friends, partner) think about your SMBG use?
   a. Have you ever felt **embarrassed about using your SMBG device**? Why? How do you deal with those feelings?

Theme 3: Facilitators and Barriers to SMBG:

10. What **features of your SMBG do you find most helpful**? Is there anything you wish it could do that it currently does not? Why?
    a. Can you give us specific examples?
11. What **challenges** (if any) have you experienced **with using your SMBG** device? How did you address them?
12. Which factors (if any) **motivate** you to use/continue to use an SMBG device?
13. How do you feel about the **results/readings from your SMBG device?**
    [Probe]: Do you feel confident or trust the readings you get from your SMBG device?
14. What factors would **influence your decision to use/not use a CGM when the study ends** [Note to interviewer: remind participants in Arm 3 that they will receive 2 CGMs after the study]?

Theme 4: Relationships:

15. Is there anyone else besides yourself, **who is involved in your diabetes management**?
    [Probe]: Such as, parents, siblings, teachers, partners? What is their role?
16. How do you feel about sharing your **glucose data with others**, such as your parents or doctors? Does it make you feel uncomfortable or empowered?
17. How would you describe your **relationship with your doctor/nurse** with respect to diabetes management?
18. How has using **SMBG device affected your relationship with your doctor/nurse?**
    [Probe]: Have you noticed any changes in how you communicate with your doctor/nurse?

Closing statement:

Before closing the session, is there **anything else you would like to add that is important to consider that we did not cover in this discussion?**

We have come to the end of the discussion. Thank you again for your time, feedback and participation. Your input is very important for us and will be valuable for future considerations regarding diabetes self-management. Please do not hesitate to contact me if you have any other questions about the study or our discussion today.

[END RECORDING]

Focus Group Discussion (FGD) Guide: Caregivers (Arm 1)

Theme 1: Knowledge and perceptions around diabetes management and CGMs:

1. What comes to your mind when you hear the word **diabetes management or diabetes control**?

2. What was your **understanding of CGMs before your child's participation** in this study?
   [Probe: Did you know about CGMs before the study? Source of information?]

3. What were your **initial thoughts/feelings** about your child using CGMs?
   [Probe]: Excited, curious, unsure…

4. What is your **current understanding of CGM** and their use in diabetes management?

Theme 2: Lived experiences of diabetes self-management using CGMs:

5. How often does your child **wear the CGM device**?
   [Probe]: What factors affect how often they wear the CGM device?

6. How often does your child **scan the CGM device (per day, per week, etc.)?**
   [Probe]: What factors affect how often they scan the CGM device?

7. How has **using a CGM device affected your child's diabetes management and glucose control?**
   [Probe]: What impact has the CGM had on your child diabetes management/how your child manage their diabetes?

8. How has **using a CGM device affected your/your child's daily routine?**
   [Probe]: What impact has the CGM had on your lives (parents and children)?

9. What **changes** (if any) have you noticed in your child's **quality of life since using a CGM** device? And yours?

10. How do you feel while your child uses a **CGM?**
    [Probe]: How do you feel about your child wearing a CGM/scanning?

11. What do people in your life (i.e. your community, family, friends, partner) think about your child's CGM use?
    a. ONLY if directly prompted by participants: Has your child felt **embarrassed or self-conscious about using the CGM device**? How do you deal with those feelings?

Facilitators and Barriers to CGM use/SMBG:

12. What **challenges** (if any) has your child experienced **with using the CGM** device? How did you address them?

13. Which factors (if any) **motivate** your child to use/continue to use a CGM device?

14. How do you feel about the **results/readings from the CGM device?**
    [Probe]: Do you feel confident or trust the readings you get from the CGM device?

15. What **challenges** (if any) have you experienced **with using the SMBG** device? How did you address them?

16. Which factors (if any) **motivate you to use/continue to use a SMBG** device?

17. How do you feel about the **results/readings from the SMBG device?**
    [Probe]: Do you feel confident or trust the readings you get from the SMBG device?

18. What factors would influence your **decision to use/not use a CGM** (even after the study has ended)?

Relationships with HCPs:

19. How would you describe your **relationship with your doctor/nurse** with respect to diabetes management? And your child's relationship with doctors and nurses?

20. How has using a **CGM device affected your relationships with your doctor/nurse?**
    [Probe]: Have you noticed any changes in how you communicate with your doctor/nurse?

<u>Closing statement:</u>

Before closing the session, is there **anything else you would like to add that is important to consider that we did not cover in this discussion?**

We have come to the end of the discussion. Thank you again for your time, feedback and participation. Your input is very important for us and will be valuable for future considerations regarding diabetes self-management. Please do not hesitate to contact me if you have any other questions about the study or our discussion today.

[END RECORDING]

Focus Group Discussion (FGD) Guide: Caregivers (Arm 2)

Theme 1: Knowledge and perceptions around diabetes management and CGMs:

1. What comes to your mind when you hear the word **diabetes management or diabetes control**?
2. What was your **understanding of CGMs before your child's participation** in this study?
   [Probe: Did you know about CGMs before the study? Source of information?]
3. What were your **initial thoughts/feelings** about your child using CGMs?
   [Probe]: Excited, curious, unsure…
4. What is your **current understanding of CGM** and their use in diabetes management?

Theme 2: Lived experiences of diabetes self-management using CGMs:

5. How often does your child **wear the CGM device**?
   [Probe]: What factors affect how often they wear the CGM device?
6. How often does your child **scan the CGM device (per day, per week, etc.)?**
   [Probe]: What factors affect how often they scan the CGM device?
7. How has **using a CGM device affected your child's diabetes management and glucose control?**
   [Probe]: What impact has the CGM had on your child diabetes management/how your child manage their diabetes?
8. How has **using a CGM device affected your/your child's daily routine?**
   [Probe]: What impact has the CGM had on your lives (parents and children)?
9. What **changes** (if any) have you noticed in your child's **quality of life since using a CGM** device? And yours?
10. How do you feel while your child uses a **CGM?**
    [Probe]: How do you feel about your child wearing a CGM/scanning?
11. What do people in your life (i.e. your community, family, friends, partner) think about your child's CGM use?
    a. ONLY if directly prompted by participants: Has your child felt **embarrassed or self-conscious about using the CGM device**? How do you deal with those feelings?

Theme 3: Facilitators and Barriers to CGM use/SMBG:

12. What **challenges** (if any) has your child experienced **with using the CGM** device? How did you address them?
13. Which factors (if any) **motivate** your child to use/continue to use a CGM device?
14. How do you feel about the **results/readings from the CGM device?**
    [Probe]: Do you feel confident or trust the readings you get from the CGM device?
15. What **challenges** (if any) have you experienced **with using the SMBG** device? How did you address them?
16. Which factors (if any) **motivate you to use/continue to use a SMBG** device?
17. How do you feel about the **results/readings from the SMBG device?**
    [Probe]: Do you feel confident or trust the readings you get from the SMBG device?

18. What factors would influence your **decision to use/not use a CGM** (even after the study has ended)?

Relationships with HCPs:

19. How would you describe your **relationship with your doctor/nurse** with respect to diabetes management? And your child's relationship with doctors and nurses?
20. How has using a **CGM device affected your relationships with your doctor/nurse?**
    [Probe]: Have you noticed any changes in how you communicate with your doctor/nurse?

<u>Closing statement:</u>

Before closing the session, is there **anything else you would like to add that is important to consider that we did not cover in this discussion?**
We have come to the end of the discussion. Thank you again for your time, feedback and participation. Your input is very important for us and will be valuable for future considerations regarding diabetes self-management. Please do not hesitate to contact me if you have any other questions about the study or our discussion today.

[END RECORDING]

Focus Group Discussion (FGD) Guide: Caregivers (Arm 3)

Theme 1: Knowledge and perceptions around diabetes management and CGMs:
What comes to your mind when you hear the word **diabetes management or diabetes control**?

1. What was your **understanding of CGMs before your participation** in this study? [Probe: Did you know about CGMs before the study? Source of information?]
2. What were your **initial thoughts/feelings** about your child using CGMs? [Probe]: Excited, curious, unsure…
3. What is your **current understanding of CGMs** and their use in diabetes management?

Theme 2: Lived experiences of diabetes self-management using a self-monitoring blood glucose (SMBG) device (i.e. a glucometer):

4. How often does your child **check his/her blood glucose using a glucometer/VivaChek**?
   [Probe]: What factors affect how often do they check/test their blood glucose?
5. How has **using a glucometer affected your child diabetes management and glucose control?**
6. How has **using a glucometer affected your daily routine/your life? And your child's?**
   [Probe]: What impact has the CGM had on your life? And your child's?
7. What do people in your life (i.e. your **community, family, friends, partner**) think about your child using a glucometer? ONLY if directly prompted by participants: Has your child ever felt **embarrassed or self-conscious about using your glucometer**? How do you deal with those feelings?

Theme 3: Facilitators and Barriers to SMBG:

8. What **challenges** (if any) has your child experienced **with using a glucometer**? How did you address them?
9. Which factors (if any) **motivate** your child to use/continue to use a glucometer?
10. Which factors (if any) motivate your child to use/continue to use a glucometer?
11. How do you feel about the **results/readings from the glucometer?**
    [Probe]: Do you feel confident or trust the readings you get from the glucometer?
12. What factors would influence your child's **decision to use/not use a CGM** (even after the study has ended)?

Relationships with HCPs:

13. How would you describe your **relationship with your doctor/nurse** with respect to diabetes management? And your child's relationship with doctors and nurses?

14. How has using **a SMBG device/glucometer** affected your relationship with your healthcare provider or care team? Have you noticed any changes in how you

communicate with your doctor/nurse? And your child's relationship with healthcare providers or care team?

<u>Closing statement:</u>

Before closing the session, is there **anything else you would like to add that is important to consider that we did not cover in this discussion?**

We have come to the end of the discussion. Thank you again for your time, feedback and participation. Your input is very important for us and will be valuable for future considerations regarding diabetes self-management. Please do not hesitate to contact me if you have any other questions about the study or our discussion today.

[END RECORDING]

Semi-structured Interview (SSI) Guide: Healthcare Providers

THEME 1. Current perceptions and attitudes towards CGMs.

1. What were your **initial perceptions** about the use of CGMs for diabetes management? (Probe: before this study)
2. What are your current **perceptions/views towards the use of CGMs** in your clinic?
    a. Can you describe your **experiences** while managing patients using the CGM? (for example, overall experience during the whole study, interesting/specific cases etc.)
    b. How do your **colleagues** view the use of the CGMs in managing patient care?
    c. How do **participants** view the use of the CGMs in monitoring their blood glucose?
    d. How do patients typically respond when **first using the CGM**?

THEME 2. Potential benefits of CGM use.

3. What do you think are the **potential benefits** of CGM use for your patients, if any?
    a. Could you give a specific example for each benefit?
    b. What specific **outcomes** have you observed, or are you **expecting to observe** by the end of the study, that demonstrate these benefits?

THEME 3. Challenges or barriers in implementing CGM use.

4. What **challenges or barriers** do you anticipate in implementing CGM use in your clinic, if any (outside the study)?
    a. Can you share a specific moment where you faced a **challenge using the CGM** (besides any challenges related to the implementation of the study)?
    b. Can you share a specific moment where your **patient faced a challenge using the CGM**?
    c. What do you believe are the **main issues/hurdles** in adopting CGMs more broadly in South Africa? (for example, access, logistics, cost, willingness to use, manufacturers, technology...)

THEME 4. Ease of CGM use.

5. Based on your experience, what is your overall impression on the **ease of use of CGMs** for healthcare providers?
    a. What features of CGMs do you find most user-friendly?
    b. And those more challenging?
6. What is your overall impression on the **ease of use of CGMs** for your patients?
    a. What features of CGMs do you think are most user-friendly for your patients?
    b. And those more challenging?

THEME 5. Support and training for successful CGM use.

7. What kind of **support and training** do you think is necessary for **health care providers** to successfully use CGMs?
    a. What specific types of training have you received to support patients?
    b. Which specific training would have been beneficial to have received by you, to support your patients better?
8. What kind of **support and training** do you think is necessary for **patients** to successfully use CGMs?
    a. Which additional materials (video, brochures etc.) would support patients?
    b. How do they usually obtain information about CGMs (Internet, friends and relatives, wait for clinic days, call the clinic etc.)?

THEME 6. Policies or guidelines for CGM integration.

9. What **policies or guidelines** do you think need to be in place to ensure the successful integration of CGMs into the public health sector?
    a. What kind of **factors** would facilitate easier adoption of CGMs in your country?
    b. What kind of **policy changes** would facilitate easier adoption of CGMs in your country?

THEME 7. Impact of CGM cost on public health integration.

10. How do you think the **cost of CGMs** will impact the feasibility and acceptability of integrating them into the public health sector?
    a. How have **cost considerations affected** decisions to wear/not wear CGMs for the participants in the study?
    b. What would be the **ideal pricing model** for CGMs to maximize accessibility? (maximum cost, co-payment strategies, government funded...)
    c. In your view, what do you think **patients would find as acceptable to pay** for CGMs (every 14 days)? Over what period of time?

THEME 8. Additional research or evidence needed.

11. What **additional research** or evidence do you think is necessary to support the integration of CGMs into the public health sector?
    a. What **unanswered questions** do you have about CGMs that additional research could help resolve?
    b. What type of **evidence would most influence policymakers, Department of Health and other key stakeholders** to support broader CGM use?

Closing statement:

Before closing the session, is there **anything else you would like to add that is important to consider that we did not cover in this discussion?**

We have come to the end of the discussion. Thank you again for your time, feedback and participation. Please do not hesitate to contact me if you have any other questions about the study or our discussion today.

[END RECORDING]
