# Supplementary material for: Exploring the feasibility and acceptability of Continuous Glucose Monitoring among people with type 1 diabetes and healthcare providers in South Africa’s public sector: A qualitative study
Source: PLoS One. 2026 Jul 16;21(7):e0352590. doi: 10.1371/journal.pone.0352590 (PMC13374883; doi:10.1371/journal.pone.0352590)
Supplement: S2 File — (PDF) [file pone.0352590.s002.pdf]

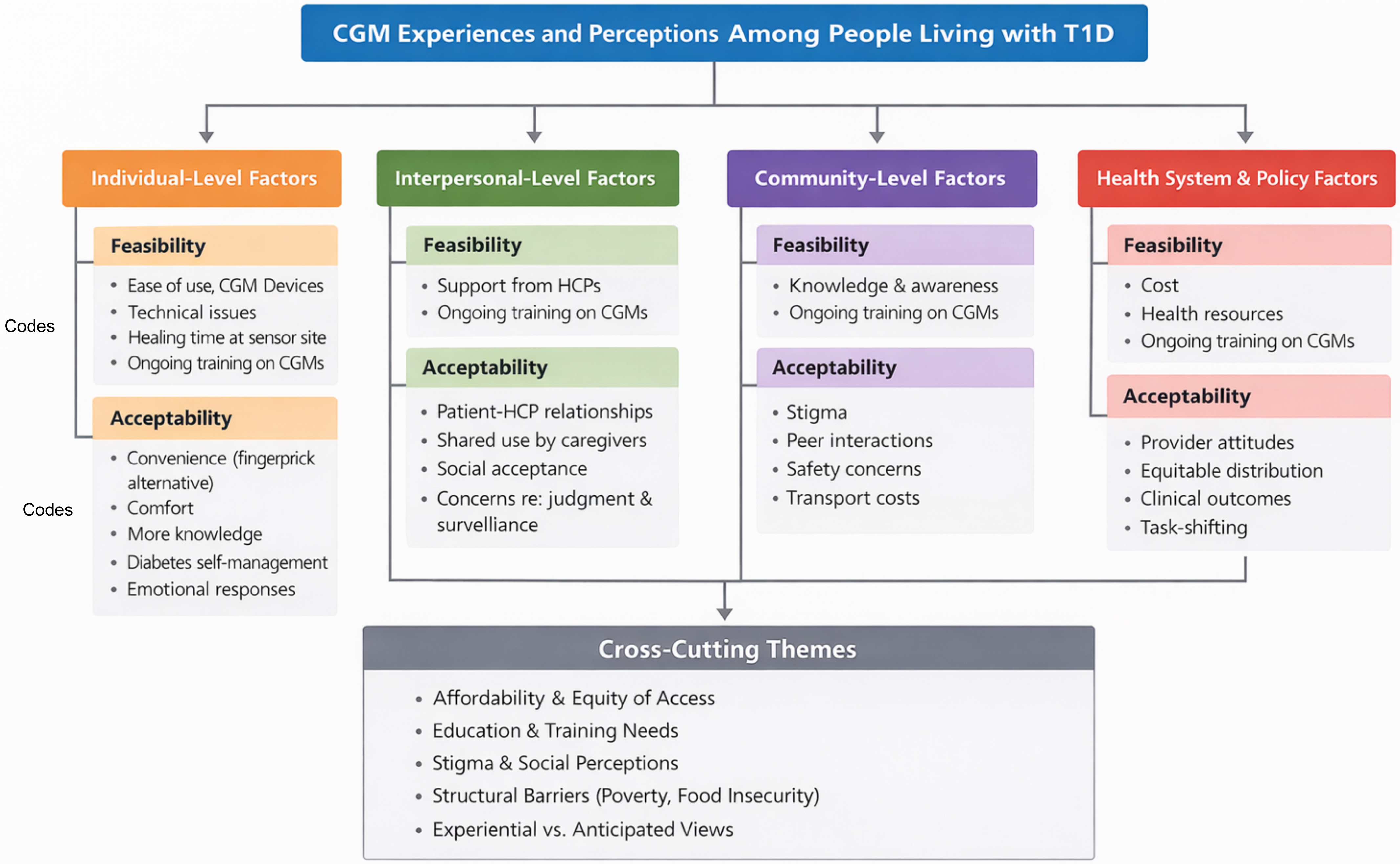
CGM Experiences and Perceptions Among People Living with T1D
Individual-Level Factors
Feasibility
Ease of use, CGM Devices
Technical issues
Healing time at sensor site
Ongoing training on CGMs
Acceptability
Convenience (fingerprick alternative)
Comfort
More knowledge
Diabetes self-management
Emotional responses
Codes
Codes
Interpersonal-Level Factors
Feasibility
Support from HCPs
Ongoing training on CGMs
Acceptability
Patient-HCP relationships
Shared use by caregivers
Social acceptance
Concerns re: judgment & surveillance
Community-Level Factors
Feasibility
Knowledge & awareness
Ongoing training on CGMs
Acceptability
Stigma
Peer interactions
Safety concerns
Transport costs
Health System & Policy Factors
Feasibility
Cost
Health resources
Ongoing training on CGMs
Acceptability
Provider attitudes
Equitable distribution
Clinical outcomes
Task-shifting
Cross-Cutting Themes
Affordability & Equity of Access
Education & Training Needs
Stigma & Social Perceptions
Structural Barriers (Poverty, Food Insecurity)
Experiential vs. Anticipated Views
